# Searching for early breast cancer biomarkers by serum protein profiling of pre-diagnostic serum; a nested case-control study

**DOI:** 10.1186/1471-2407-11-381

**Published:** 2011-08-26

**Authors:** Annemieke WJ Opstal-van Winden, Esmeralda JM Krop, Monica H Kåredal, Marie-Christine W Gast, Christian H Lindh, Marina C Jeppsson, Bo AG Jönsson, Diederick E Grobbee, Petra HM Peeters, Jos H Beijnen, Carla H van Gils, Roel CH Vermeulen

**Affiliations:** 1 Julius Center for Health Sciences and Primary Care, University Medical Center Utrecht, Universiteitsweg 100, 3584 CG Utrecht, The Netherlands; 2 Department of Pharmacy & Pharmacology, The Netherlands Cancer Institute/Slotervaart Hospital, Louwesweg 6, 1066 EC Amsterdam, The Netherlands; 3 Institute for Risk Assessment Sciences, Utrecht University, Jenalaan 18D, 3584 CK Utrecht, The Netherlands; 4 Department of Occupational and Environmental Medicine, Lund University, SE- 221 85 Lund Sweden; 5 Central Hospital Pharmacy, Escamplaan 900, 2547 EX The Hague, The Netherlands; 6 Department of Epidemiology, Public Health and Primary Care, Faculty of Medicine, Imperial College Norfolk Place, London W2 1PG, UK; 7 Faculty of Science, Department of Pharmaceutical Sciences, Division of Biomedical Analysis, Section of Drug Toxicology, Utrecht University, Sorbonnelaan 16, 3584 CA Utrecht, The Netherlands

**Keywords:** Biomarkers, Breast cancer, Early diagnosis, 2D-nanoLC-MS/MS, Prospective, Proteomics, SELDI-TOF MS

## Abstract

**Background:**

Serum protein profiles have been investigated frequently to discover early biomarkers for breast cancer. So far, these studies used biological samples collected *at *or *after *diagnosis. This may limit these studies' value in the search for cancer biomarkers because of the often advanced tumor stage, and consequently risk of reverse causality. We present for the first time pre-diagnostic serum protein profiles in relation to breast cancer, using the Prospect-EPIC (European Prospective Investigation into Cancer and nutrition) cohort.

**Methods:**

In a nested case-control design we compared 68 women diagnosed with breast cancer within three years after enrollment, with 68 matched controls for differences in serum protein profiles. All samples were analyzed with SELDI-TOF MS (surface enhanced laser desorption/ionization time-of-flight mass spectrometry). In a subset of 20 case-control pairs, the serum proteome was identified and relatively quantified using isobaric Tags for Relative and Absolute Quantification (iTRAQ) and online two-dimensional nano-liquid chromatography coupled with tandem MS (2D-nanoLC-MS/MS).

**Results:**

Two SELDI-TOF MS peaks with m/z 3323 and 8939, which probably represent doubly charged apolipoprotein C-I and C3a des-arginine anaphylatoxin (C3a_desArg_), were higher in pre-diagnostic breast cancer serum (p = 0.02 and p = 0.06, respectively). With 2D-nanoLC-MS/MS, afamin, apolipoprotein E and isoform 1 of inter-alpha trypsin inhibitor heavy chain H4 (ITIH4) were found to be higher in pre-diagnostic breast cancer (p < 0.05), while alpha-2-macroglobulin and ceruloplasmin were lower (p < 0.05). C3a_desArg _and ITIH4 have previously been related to the presence of symptomatic and/or mammographically detectable breast cancer.

**Conclusions:**

We show that serum protein profiles are already altered up to three years before breast cancer detection.

## Background

Early diagnosis of breast cancer by mammography is one of the most important factors contributing to the successful treatment of breast cancer. Further improvement of early diagnosis might be possible with the use of blood-based biomarkers. Such markers could indicate the presence of a breast tumour already in an early stage, preferably even before the lesion is visual on a mammogram. This would be particularly relevant for young women for whom mammographic screening is less effective due to lower sensitivity (25 to 59%) [1]. Although the addition of magnetic resonance imaging (MRI) to mammography could improve sensitivity [1], a blood test would be less expensive and easier to perform on a large scale.

Many studies have been executed in an attempt to find such early breast cancer biomarkers, for example using surface enhanced laser desorption/ionization time-of-flight mass spectrometry (SELDI-TOF MS) [2-9]. Several proteins in the blood were indeed found to be related to the presence of breast cancer [2-9]. However, only few of these proteins were reported to be discriminative for breast cancer in more than one study, and even then, some proteins found to be higher in patients in one study, were found to be lower in another study [2-9]. These discrepancies may be caused by differences between cases and controls in collection, processing and storage of their blood samples, both within and between studies [10-16]. On the other hand, it cannot be excluded that findings were simply due to chance.

Until now, all studies, except one by Pitteri et al. [17], used biological samples collected *at *or *after *diagnosis of breast cancer, and thus findings may reflect consequences rather than predictors of malignancy. Thus, it remains unclear whether these proteins are able to identify women with a breast lesion which is not yet visible on a mammogram and does not induce clinical symptoms yet. Pitteri et al. [17] previously investigated *plasma *samples prospectively collected in the Women's Health Initiative Observational Study. Epidermal Growth Factor Receptor (EGFR) was found to be increased in plasma samples collected 17 months before breast cancer diagnosis. In the present study we performed *serum *protein profiling of breast cancer samples for the first time in a nested case-control study. For this we used the Prospect-EPIC (European Prospective Investigation into Cancer and nutrition) cohort [18], where at study enrollment blood samples of approximately 17,000 healthy women were collected and stored. For the current study we selected those women who were diagnosed with breast cancer within 3 years after enrollment in the cohort. Pre-diagnostic protein profiles of their serum samples, taken at enrollment, were compared to those of matched controls who remained healthy.

Our first aim was to assess whether previously reported proteins are also discriminative in serum samples taken up to three years *before *breast cancer diagnosis. We also set out to discover new discriminating proteins. To this end, we used SELDI-TOF MS that has the possibility to measure multiple proteins simultaneously in a high-throughput fashion. Next, in a subset of the case-control pairs, we analyzed the serum protein profiles with isobaric Tags for Relative and Absolute Quantification (iTRAQ)-labeling, and two-dimensional online nano-liquid chromatography coupled with tandem mass spectrometry (2D-nanoLC-MS/MS), by which the detected proteins are relatively quantified and immediately identified. SELDI-TOF MS and 2D-nanoLC-MS/MS cover different mass ranges and therefore are able to detect different proteins.

In summary, we set out to find new proteins as well as to test previously detected proteins in patients still free of symptomatic breast cancer.

## Methods

### Study population

We performed a case-control study nested within the Prospect-EPIC cohort. Prospect-EPIC is one of the two Dutch cohorts participating in the European Prospective Investigation into Cancer and nutrition, which includes ten European countries. From 1993 to 1997, 17,357 women from Utrecht and vicinity, aged 50-69 years, enrolled in this cohort through the national population-based breast cancer screening program [18]. Women filled out an extensive food frequency questionnaire and a general questionnaire. The latter contained questions on demographic characteristics, medical history, lifestyle characteristics, and risk factors for cancer and other chronic diseases [18,19].

Prospect-EPIC participants also donated a blood sample. Blood collection, processing and storage were performed following a strict protocol. After collection, blood samples were stored in a climate controlled refrigerator at 5°C overnight. The next day blood samples were centrifuged at 1500 g for 20 minutes. After centrifuging, the serum was put in 0.5 ml straws. These straws were stored in a -86°C freezer until they were transported to liquid nitrogen tanks (-196°C), where they have been stored since then.

Participants were followed for vital and health status. Information on dates of death and migration was obtained through the municipal registries. Causes of death were obtained from the Central Bureau for Statistics (CBS). Through yearly linkage with the regional and national cancer registries information about cancer incidence and stage of disease at diagnosis (tumor behavior, tumor size, lymph node involvement and metastasis) was obtained [18]. Until December 31^st ^2006, 687 women were diagnosed with breast cancer in the Prospect-EPIC cohort. All participants signed an informed consent and the study was approved by the Institutional Review Board of the University Medical Center Utrecht.

For the current study we selected women who were diagnosed with breast cancer within three years after enrollment into the cohort, and who were postmenopausal at enrollment (no menstrual periods in last 12 months). Women were excluded if they had had cancer before, were suffering from diabetes, were current smokers, or were currently using oral contraceptives, or menopausal hormone therapy (HT). This was done to obtain a homogeneous group with respect to hormone levels, smoking status, and metabolic status, because these factors (may) influence serum protein profiles [20]. Sixty-eight women were eventually included as a case. Controls were participants of the same cohort. We matched each case with one postmenopausal control that remained free of breast cancer up to the time the case was diagnosed. Additional matching factors were age at enrollment (± 1 year) and date of enrollment (± 1/2 year). For controls the same exclusion criteria were applied as for cases. Differences between cases and controls, and between samples of cases and controls, were tested with independent samples T test for normally distributed continuous variables, with Mann-Whitney U test for other continuous variables, and Pearson Chi-Square for categorical variables.

### SELDI-TOF MS analysis

We performed serum protein profiling on immobilized metal affinity capture (IMAC30) ProteinChip arrays (Bio-Rad Labs, Hercules, Ca, USA) activated with nickel as described in our previous study [9]. The total sample set was analyzed in duplicate, in three separate batches, within two weeks time. Duplicates were analyzed within the same batch, but on different arrays, to correct for inter-array variability. Cases and controls were evenly, and randomly, distributed over the three batches. Samples in one batch were prepared and applied to the arrays, followed by detection of the proteins bound to the arrays with SELDI-TOF MS, on the same day. SELDI-TOF MS was performed using the PBS-IIC ProteinChip Reader (Bio-Rad Labs). See Additional file 1 for settings of the ProteinChip Reader.

Since analyzing samples in different batches, on different days, introduces inter-batch variation [16,21,22], spectra were processed per batch. For this, we used the ProteinChip Software package, version 3.1 (Bio-Rad Labs). Spectra in which normalization revealed too low or too high total ion current were excluded from further analysis. The cases and controls matched with these subjects were also excluded from the paired analyses. Subsequently, the Biomarker Wizard (BMW) software application (Bio-Rad Labs) was used to detect peaks. This was performed in each batch separately. See Additional file 1 for way of processing the spectra and for the settings for peak detection.

### SELDI-TOF MS data analysis

Peak information from all acquired spectra was exported from the ProteinChip Software to SPSS 15.0 for statistical analysis. First, we estimated the reproducibility of the duplicates, by calculating the median coefficient of variance (CV) for each detected peak, in cases and controls together. The averaged intensities of the peaks with the same mass in the duplicate spectra of a subject were used for further analysis. To be able to merge peak intensity data of the three batches, averaged peak intensities were first Z-log-transformed per batch [23].

Paired samples T tests were used to test if the mean Z-log-transformed peak intensities in the pre-diagnostic breast cancer serum samples were statistically significantly different from those in the controls samples. We performed correction for multiple testing, using the False Discovery Rate (FDR) method suggested by Benjamin and Hochberg. The FDR controls the expected proportion of falsely rejected hypothesis [24]. We chose 10% as an acceptable proportion of false positive results (q-value = 0.10). We also investigated whether any significant relation could be explained by any of the subject characteristics other than breast cancer status. To this end, bivariate conditional logistic regression analyses were performed including the peak intensity (continuous) and one of the following characteristics: Body Mass Index (BMI), former use of oral contraceptives, former use of HT, number of children, smoking habits, alcohol consumption, blood sample's time in refrigerator between blood collection and centrifugation, and sample's time in -86°C freezer until storage at liquid nitrogen. The adjusted odds ratios (OR) resulting from the analyses were compared with the crude breast cancer OR in relation to peak intensity. To test whether the intensities of peaks that differed between cases and controls, also differed between cases that were more close to diagnosis, and cases that were less close to diagnosis at moment of sample collection, we performed independent sample T tests. To this end, cases who were diagnosed based on the first mammogram after enrollment were compared to cases who had a negative first mammogram and who were diagnosed at a later moment.

### Sample preparation for 2D-nanoLC-MS/MS

We restricted the 2D-nanoLC-MS/MS analysis to 20 case-control pairs, because of costs and time restrictions. The cases included in this sub-analysis were diagnosed with breast cancer within the first 14 months after enrollment in the study.

The serum samples were depleted of the high abundant proteins albumin, IgG, antitrypsin, IgA, transferrin and haptoglobin, using the Multiple Affinity Removal Spin Cartridge (Hu-6HC, Agilent Technologies, Santa Clara, CA, USA) as described in the manufacturer's protocol. Thereafter the samples were desalted using Microcon Centrifugal Filter units (Millipore, Billerica, MA. USA). The total protein content of the depleted sera was determined using a protein assay kit (BCA™, Pierce, Thermo Scientific, Rockfort, IL, USA). The proteins (50 μg per sample) were reduced using tris(2-carboxyethyl)phosphine, alkylated using iodoacetamide and then trypsin digested (Roche Diagnostics Gmbh, Mannhein, Germany) overnight and evaporated to dryness using a SpeedVac. Peptides were labeled with 4-plex iTRAQ reagents (iTRAQ reagent kit-plasma, Applied Biosystems, Foster City, CA, USA) according to the instructions of the manufacturer.

Two case-control pairs were labeled with different isobaric tags in each iTRAQ-labeling set. The first case was labeled with tag114 and the matching control with tag115, the next case was labeled with tag116 and the matching control with tag117. The 4 labeled samples were finally pooled into a new sample tube. A total of 10 iTRAQ-labeled sample sets consisting of two case-control pairs were generated.

### 2D-nanoLC-MS/MS analysis

The 10 iTRAQ-labeled sample sets were analyzed using quadrupole-time-of-flight mass spectrometer (QSTAR pulsar; Applied Biosystems), equipped with a nanoelectrospray source (Proxeon, Odense, Denmark), and connected to a 2D-nanoLC system equipped with a capillary and nano pump (1100 series; Agilent Technologies). See Additional file 2 for details about the used columns and mobile phases. The LC system was coupled on-line to a fused-silica PicoTip (50 μm i.d. × 360 μm o.d. × 8 μm tip; New Objective, Woburn, MA, USA). Details about acquisition and calibration are also described in Additional file 2.

### 2D-nanoLC-MS/MS data analysis

Protein identifications and quantifications were performed using Protein Pilot 1.0 (Applied Biosystems) in which the paragon search algorithm was applied. Proteins were searched against the IPI human protein database (IPI human v3.40) downloaded from http://www.ebi.ac.uk[25]. See Additional file 3 for details on search parameters and data processing.

In some runs, some peptides were unusable for quantification due to an artificial low signal of the signature ions or because the peptide sequence was shared by other proteins. In those cases the peptides were excluded from quantification. No iTRAQ ratio was calculated if there was not one usable peptide left. If only one peptide was usable for quantification of a protein then no error factor (EF) was calculated. A case-control pair was excluded when no ratio and/or EF could be calculated for this pair. Only proteins that could be measured in at least 14 of the 20 case-control pairs were selected for further analysis.

The ratios and the EFs for a protein, in the different pairs, were used to model a random effect model. We used the random effect model since we assumed heterogeneity between the ratios of the different pairs that is partly based on variation by coincidence, but also on true variation between the pairs. The random effect model resulted in a weighted mean ratio with a 95% confidence interval (95%CI) for every protein. We also applied correction for multiple testing using de FDR method on these results. We again choose 10% as an acceptable proportion of false positive results.

## Results

### Study population

Characteristics of the total study population are presented in Table 1. About half of both cases and controls used oral contraceptives in the past, but the cases used them for a longer period of time than the controls (median: 10 years and 4.5 years, respectively; p-value 0.018). Cases were somewhat more often nulliparous (15%) than controls (7%), and among women with children, controls had more children than the cases; 3 and 2 (median), respectively, although not statistically significantly. About half of both cases and controls had smoked in the past, for about 8 and 4 pack-years (median), respectively (p = 0.187). Characteristics of the serum samples and the sample collection are listed in Table 2. There was no difference between cases and controls regarding sample collection and storage. Characteristics of the subjects in the subset (analyzed by 2D-nanoLC-MS/MS), and of their serum samples, are shown in Additional file 4 and 5.

**Table 1:** Study population characteristics

	Cases (*n *= 68)	Controls (*n *= 68)	**P-value**^††^
**Age at enrollment **(years)			
Mean (SD)	60.2 (5.6)	60.3 (5.7)	0.966
**BMI**			
Mean (SD)	26.6 (3.1)	26.3 (3.6)	0.603
Missing	1	-	
**Use of oral contraceptives**, *n *(%)			
No, but used to in the past	36 (52.9)	40 (58.8)	0.490
No, never	32 (47.1)	28 (41.2)	
Duration of oral contraceptives use* (years)			
Median (IQR)	10.0 (4.3-15.8)	4.5 (2.0-10.0)	0.018
**Use of HT**, *n *(%)			
No, but used to in the past	7 (10.3)	6 (8.8)	0.771
No, never	61 (89.7)	62 (91.2)	
Duration of HT use* (years)			
Median (IQR)	1.0 (1.0-8.0)	2.0 (1.0-10.0)	0.273
**Ovariectomy**, *n *(%)			
Both ovaries removed	5 (7.4)	3 (4.5)	0.479
Missing	-	1	
**Parity**, *n *(%)			
Nulliparous	10 (14.7)	5 (7.4)	0.171
**Number of children**^†^			
Median (IQR)	2.0 (2.0-3.0)	3.0 (2.0-3.0)	0.100
**Smoking**, *n *(%)			
No, but used to in the past	31 (45.6)	34 (50.0)	0.607
No, never	37 (54.4)	34 (50.0)	
**Pack-years smoking until stop date**^‡^			
Median (IQR)	7.9 (1.9-16.4)	4.1 (1.4-10.2)	0.187
Missing	1	3	
**Alcohol intake **(g/day)^§^			
Median (IQR)	2.0 (0.2-7.2)	2.5 (0.2-8.4)	0.638
**Use of medicines, minerals or vitamins**^#^, *n *(%)			
Yes	46 (67.6)	44 (64.7)	
No	22 (32.4)	24 (35.3)	0.717
Time since last meal and/or drink** (min)			
Median (IQR)	108 (87-137)	116 (88-137)	0.651

SD: standard deviation; BMI: body mass index; IQR: inter-quartile range; HT: menopausal hormone therapy; * Among former oral contraceptives/HT users; ^† ^Among women with children; ^‡ ^Among former smokers; ^§ ^Energy-adjusted alcohol intake at enrollment; ^# ^In last week before blood collection; ** At moment of blood collection; ^†† ^Independent samples T test for age and BMI, Mann-Whitney U test for other continuous variables, and Pearson Chi-Square test for categorical variables.

**Table 2:** Characteristics of the serum samples

	Cases (*n *= 68)	Controls (*n *= 68)	P-value
Serum sample storage duration* (years)			
Mean (SD)	11.2 (1.1)	11.2 (1.1)	0.900^§^
**Hours in refrigerator**^†^			
Median (IQR)	22 (21-23)	22 (20-23)	0.845^#^
**Days at -86°C**^‡^			
Median (IQR)	8 (6-11)	7 (5-11)	0.429^#^

SD: standard deviation; IQR: inter-quartile range; * Until date of experiment; ^† ^Between collection and centrifugation; ^‡ ^Between centrifugation and storage at liquid nitrogen; ^§ ^Independent samples T test; ^# ^Mann-Whitney U test.

Breast cancer was diagnosed after a median time of 21.3 months (inter-quartile range (IQR): 0.7-26.6) after enrollment. More than 80% of the cases had an invasive tumor. More than half of the invasive tumors were diagnosed in Stage I and a quarter of the invasive tumors were diagnosed in Stage IIA. Only one tumor was diagnosed in Stage IIIA. The invasive tumors were more or less equally distributed over the three size categories (>0.1-1 cm, 1-2 cm and >2 cm). In almost 30% of the invasive tumors, lymph nodes were involved. None of the cases was diagnosed with distant metastasis. We reported the pathologically determined tumor size and lymph node involvement unless this was unknown; in that case we reported the clinically determined stage. Cases in the subset analyzed by 2D-nanoLC-MS/MS were diagnosed 0.9 months (median) (IQR: 0.6-7.5) after enrollment. Two of the 20 cases were diagnosed with carcinoma in situ. Two thirds of the invasive tumors were diagnosed in Stage I and almost a quarter in Stage IIA, the remaining tumors were diagnosed in Stage IIB. Half of the invasive tumors were sized <1 cm, and in only three invasive tumors lymph nodes were involved.

### Peaks detected with SELDI-TOF MS

After normalization, 25 of the 272 spectra (68 cases and 68 controls in duplicate) had to be eliminated from the analysis. These outliers included 12 spectra of cases and 13 spectra of controls. Of one case and two controls both spectra (duplicates) had to be eliminated. With the BMW software application, in total 47 different peaks were auto-detected in the three batches. Twenty-two of these peaks were present with an S/N >2 in at least 50% of the spectra in each batch. The median CV's of these peaks varied between 12% and 35%.

**Figure 1:**
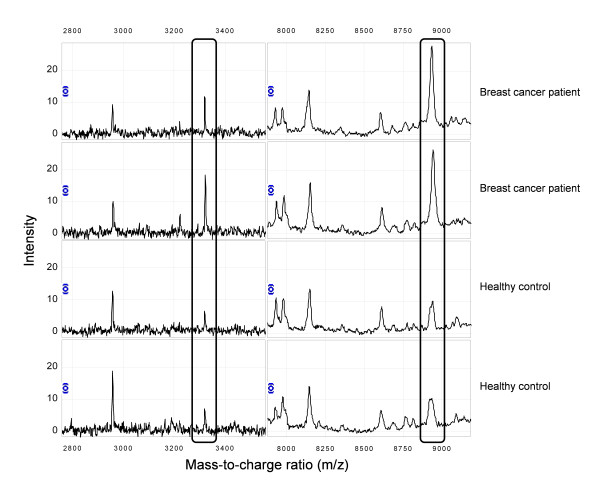
**Difference in protein expression of *m/z *3323 and *m/z *8938, detected with SELDI-TOF MS, between breast cancer cases and healthy controls**.

The intensity of a peak with mass-to-charge ratio (*m/z*) 3323 was statistically significantly higher in pre-diagnostic breast cancer serum samples than in serum samples of controls (p = 0.02). The intensity of a peak with *m/z *8938 was borderline statistically significantly higher in cases than in controls (p = 0.06) (Figure 1). No statistically significant relations were found between the intensities of the other detected peaks and the early presence of breast cancer. Correction for multiple testing revealed that none of the detected peaks had less than 10% chance to be a false positive finding. The 22 detected peaks ordered by their *m/z*, together with their mean Z-log-transformed peak intensities in cases and controls, and the results of the paired T test are listed in Table 3.

**Table 3:** The Z-log-transformed intensities of the peaks detected with SELDI-TOF MS, ordered by their *m/z*.

	Cases (*n *= 65)	Controls (*n *= 65)		Paired T test
***M/z***	***Mean*** ***Z-log-transformed*** ***intensity (SD)***	***Mean*** ***Z-log-transformed*** ***intensity (SD)***	***Intensity in cases*** ***vs. controls***	***p-value***

2958	0.06 (0.95)	-0.03 (1.02)	-	.61
3323	0.21 (0.98)	-0.19 (0.97)	Higher	.02
3888	0.12 (1.00)	-0.13 (0.99)	-	.18
4649	0.03 (0.95)	-0.02 (1.00)	-	.80
4824	-0.09 (0.85)	0.13 (1.11)	-	.21
5343	0.09 (0.87)	-0.05 (1.08)	-	.43
5911	0.00 (0.76)	0.03 (1.16)	-	.86
6117	0.03 (0.90)	-0.01 (1.06)	-	.81
6439	0.09 (0.99)	-0.11 (1.01)	-	.27
6637	0.11 (0.97)	-0.10 (1.03)	-	.23
6842	0.08 (1.01)	-0.07 (1.00)	-	.43
6948	-0.12 (1.01)	0.14 (0.93)	-	.15
7476	-0.04 (0.94)	0.05 (1.06)	-	.62
7772	0.06 (0.95)	-0.08 (1.05)	-	.45
7978	0.11 (0.97)	-0.12 (1.02)	-	.21
8148	0.11 (0.97)	-0.12 (1.02)	-	.21
8609	-0.04 (1.02)	0.07 (0.97)	-	.55
8938	0.19 (1.02)	-0.14 (0.94)	Higher	.06
9294	0.03 (0.89)	0.00 (1.03)	-	.88
9427	0.15 (1.09)	-0.17 (0.88)	-	.08
9501	0.06 (0.87)	-0.05 (1.06)	-	.54
13892	-0.08 (0.97)	0.12 (0.95)	-	.26

*M/z*: mass-to-charge ratio; SD: standard deviation; -: No statistically significant difference in intensity

Bivariate conditional logistic regression analysis revealed that the relations between *m/z *3323 and breast cancer, and *m/z *8938 and breast cancer, were independent of BMI, oral contraceptives use, HT use, number of children, smoking habits, alcohol intake, duration of blood sample in refrigerator between collection and centrifugation, or serum sample storage duration at -86°C before storage at liquid nitrogen (data not shown).

Twenty-three cases were diagnosed based on the first screening after enrollment, 43 cases had a negative mammogram at first screening and were diagnosed at a later moment. The mean Z-log-transformed intensity of *m/z *3323 was not different between the early breast cancer cases and the very early breast cancer cases (0.22 (SD:0.96) and 0.21 (SD:1.00), respectively; p = 0,99). The mean Z-log-transformed intensity of *m/z *8938 was somewhat higher in the early breast cancer cases, compared to the very early breast cancer cases, although not statistically significantly (0.23 (SD:0.86) and 0.16 (SD:1.11), respectively; p = 0.79).

### Identities of the SELDI-TOF MS peaks

Based on results of a previous study performed by our group [26], the peak with *m/z *3323 is likely to be doubly charged apolipoprotein C-I. We previously identified a 6.6 kDa peak as apolipoprotein C-I (molecular weight (MW): 6631 Da) by biomarker purification, in-gel tryptic digestion and peptide mapping. Its identity was confirmed with an immunoassay. In the same study, a highly correlated 3.3 kDa peak was found to be the result of double charged apolipoprotein C-I ions [26]. Although these peaks were detected on different ProteinChip arrays (CM10 cation exchange surface), this protein may also bind to the IMAC30 Ni-metal-affinity surface. An extra argument is that besides *m/z *3323, we also detected the peak representing apolipoprotein C-I itself in the current study (*m/z *6637). Although its relationship with early stage breast cancer was not statistically significant (p = 0.23), the Z-log-transformed intensities of *m/z *6637 and *m/z *3323 detected in the current study were also correlated (Pearson R^2 ^= 0.558 (p < 0.001) in the controls), as expected between a protein and its doubly charged ion.

The peak with *m/z *8938 is likely to be C3a des-arginine anaphylatoxin (C3a_desArg_) (MW: 8939 Da), based on a previous study by our group [27]. In that study a peak with *m/z *8937 was identified as C3a_desArg _by protein purification and in-gel tryptic digestion, followed by peptide mapping. The identity of the peak was confirmed by sequencing the tryptic digest peptides by quadrupole-time-of-flight MS and by an immunoassay on ProteinA beads [27].

### Proteins detected with 2D-nanoLC-MS/MS

In total, 110 different proteins were detected in the samples of the 20 cases-control pairs with 2D-nanoLC-MS/MS. For only 32 of the detected proteins, ratios and EF's could be calculated for at least 14 of the 20 case-control pairs (Table 4). Afamin, apolipoprotein E and an isoform of inter-alpha trypsin inhibitor heavy chain H4 (ITIH4) were statistically significantly higher (p < 0.05) in cases than in controls (weighted mean ratio: 1.10 (95%CI: 1.02-1.18), 1.13 (95%CI: 1.01-1.26) and 1.08 (95%CI: 1.03-1.14), respectively). Alpha-2-macroglobulin and ceruloplasmin were statistically significantly lower (p < 0.05) in cases than in controls (weighted mean ratio: 0.94 (95%CI: 0.88-1.00) and 0.94 (95%CI: 0.89-0.99), respectively). After correction for multiple testing using the FDR, ITIH4 appeared to have less than 10% chance to be a false positive finding.

**Table 4:** Proteins detected with 2D-nanoLC-MS/MS in 14 pairs or more

		Pairs*	Weighted ratio^†^		Random fixed effects model
***Protein Name***	***Function***	***n***	***Mean***	***95%CI***	***p-value***

Vitronectin		16	1.06	0.99-1.13	.07
Transthyretin		15	0.98	0.88-1.09	.71
Alpha-1B-glycoprotein		20	1.03	0.99-1.07	.17
**Alpha-2-macroglobulin**	**Proteinase inhibitor**	**20**	**0.94**	**0.88-1.00**	**.04**
**Afamin**	**Serum transport protein**	**17**	**1.10**	**1.02-1.18**	**.02**
AMBP protein		18	1.04	0.97-1.12	.26
Apolipoprotein A-I		20	1.04	0.97-1.12	.25
Apolipoprotein A-II		20	0.98	0.91-1.06	.61
Apolipoprotein A-IV		20	1.05	0.95-1.18	.32
Apolipoprotein B-100		20	1.06	0.99-1.12	.08
Complement C3 (Fragment)		20	1.02	0.98-1.06	.34
Isoform 1 of Complement factor H		18	1.02	0.97-1.07	.39
**Ceruloplasmin**	**Acute phase reactant**	**20**	**0.94**	**0.89-0.99**	**.03**
Hemopexin		20	0.96	0.90-1.02	.17
Histidine-rich glycoprotein		20	0.96	0.89-1.04	.30
Inter-alpha trypsin inhibitor heavy chain H1		16	1.00	0.94-1.07	.89
Alpha-1-acid glycoprotein 2		16	1.00	0.93-1.08	.91
Inter-alpha (Globulin) inhibitor H2		18	0.96	0.89-1.04	.33
Orosomucoid 1		20	1.06	0.98-1.14	.16
Alpha-1-antichymotrypsin		19	1.00	0.92-1.08	.94
B-factor, properdin		18	0.99	0.94-1.05	.76
Plasminogen		18	0.98	0.90-1.07	.69
Alpha-2-HS-glycoprotein		18	1.00	0.94-1.07	.88
Beta-2-glycoprotein 1		18	0.99	0.92-1.05	.66
C4B1		16	0.99	0.93-1.05	.74
Prothrombin (Fragment)		16	0.98	0.88-1.10	.76
**Apolipoprotein E**	**Lipid metabolism**	**16**	**1.13**	**1.01-1.26**	**.04**
Apolipoprotein C-I		14	1.02	0.94-1.12	.57
13 kDa protein		14	1.03	0.92-1.16	.55
Isoform LMW of Kininogen-1		18	1.06	0.98-1.14	.13
**Isoform 1 of inter-alpha trypsin inhibitor heavy chain H4**	**Acute phase reactant**	**16**	**1.08**	**1.03-1.14**	**<.01**
Vitamin D-binding protein		20	1.03	0.99-1.07	.17

95%CI: 95% confidence interval; * Number of pairs in which a ratio could be determined and a EF could be calculated; ^† ^Ratio case/control

## Discussion

We found several proteins that showed different intensities in pre-diagnostic serum samples of breast cancer cases not yet showing clinical symptoms compared to samples of healthy controls. Two proteins detected with SELDI-TOF MS, one with *m/z *3323, which is likely to be a double charged ion of apolipoprotein C-I, and another with *m/z *8938, which is likely to be C3a_desArg_, were found to be related to pre-diagnostic breast cancer. Of the proteins detected with 2D-nanoLC-MS/MS, afamin, apolipoprotein E and an isoform of ITIH4 were slightly, but significantly higher and alpha-2-macroglobulin and ceruloplasmin slightly, but significantly lower in pre-diagnostic breast cancer samples compared to control samples. Although correction for multiple testing revealed that only ITIH4 had less than 10% chance to be a false positive finding, several of the other proteins have previously been found in relation with symptomatic breast cancer. *M/z *3323, which probably represents the double charged ion of apolipoprotein C-I, showed the largest difference between cases and controls. Apolipoprotein C-I itself, detected both with SELDI-TOF MS (*m/z *6637) and 2D-nanoLC-MS/MS, showed results in the same direction, i.e. higher in cases, but not statistically significantly. In a study by Engwegen et al. [26], examining serum samples taken after diagnosis, the doubly charged ion of apolipoprotein C-I was lower in breast cancer cases, but not statistically significantly. Apolipoprotein C-I itself (6631 Da), was statistically significantly lower in breast cancer cases in that study [26]. It is striking that the same protein was found to be related with breast cancer in both studies, but in different directions. This may be due to differences in sample collection, processing and storage, but also to the differences in stage of disease of the two study populations. We included samples collected up to three years before diagnosis, while in the study by Engwegen et al. [26] samples were collected after diagnosis. Apolipoprotein C-I may be differently expressed in pre-diagnostic stages of breast cancer compared to stages visible on a mammogram and/or leading to clinical symptoms. It is also possible that the result is a chance finding.

*M/z *8938, probably representing C3a_desArg_, that we found to be higher in pre-diagnostic breast cancer samples, has been found to be related to breast cancer in several previous SELDI-TOF MS studies [2,3,6-8,28]. In the majority of these studies the protein was higher in patients compared to controls [3,6-8], but in two studies it was lower [2,9]. ITIH4 was higher in our pre-diagnostic breast cancer samples than in the control samples. This is a protein of which fragments have been frequently described in relation to symptomatic and/or mammographically detectable breast cancer [6-9,29-31]. In these studies levels of a 4.3 kDa ITIH4 fragment were found either to be significantly higher [7,30], or significantly lower [6,8,9] in breast cancer. Levels of other fragments of ITIH4, which were investigated by Villanueva et al. [29], Song et al. [30], and our own group [31], were usually found to be higher in breast cancer or were not related at all [29,30].

To our knowledge, afamin, apolipoprotein E, alpha-2-macroglobulin and ceruloplasmin have not been found before to differ between breast cancer serum samples and control serum samples in studies using SELDI-TOF MS or other profiling methods. In the 1980s however, the acute phase proteins alpha-2-macroglobulin and ceruloplasmin were already studied in relation to breast cancer, using immunoassay methods [32,33]. Serum levels of alpha-2-macroglobulin did not differ between breast cancer patients and women with benign breast disease [32]. In our study, alpha-2-macroglobulin and ceruloplasmin were both lower in pre-diagnostic breast cancer samples compared to the control samples.

It may be a limitation that we did not perform structural identification, and validation of the discriminative power in an independent validation set, of the two discriminative proteins detected with SELDI-TOF MS. However, it is very likely that these proteins are acute phase reactants, which are not cancer specific, let alone breast cancer specific. Therefore, we decided not to invest in structural identification and validation. Moreover, another similar study population was not available for validation. Nevertheless, it is very interesting that this kind of proteins is already discriminative up to three years before the diagnosis of breast cancer. Therefore, our results should not draw our attention to these specific proteins, and their potential as breast cancer biomarkers, but rather to the fact that an inflammatory process is already measurable up to three years before diagnosis, at a moment that only few tumor cells or a very small tumor may be present.

The most important strength of our study is that we investigated proteomic profiles in serum of patients with asymptomatic breast cancer (diagnosed after a median time of 21.3 months (IQR: 0.7-26.6) after enrollment). Our study population therefore is more appropriate for finding early breast cancer biomarkers than all previous studies where mostly symptomatic cases were included. The case-control design nested in a cohort of, apparently healthy screening participants also ensures that all serum samples were collected, processed and stored uniformly under strictly defined conditions, at a time when none of the participants were diagnosed with breast cancer yet. These factors have shown to be important in protein profiling studies [10-16]. In this way systematic errors due to differences in these factors between cases and controls were prevented in our study. Moreover, we were able to control for many (possible) confounding variables, by including only post-menopausal women, who never had cancer before, were not diabetic, were not current smokers, and did not currently use oral contraceptives or menopausal hormone therapy [20]. Furthermore, we could correct the results for age, BMI, past oral contraceptive and HT use, number of children, past smoking habits, alcohol intake, and several serum sample characteristics.

A limitation of our study is that, due to the strict selection criteria and the limited availability of pre-diagnostic serum samples of breast cancer cases, we were only able to include 68 case-control pairs in our study. Due to time and cost restriction, for the 2D-nanoLC-MS/MS analysis we only included the 20 cases that were diagnosed with breast cancer within the first 14 months after enrollment in the study, and their matched controls. These samples sizes are limited, but the strict selection criteria also prevented bias and confounding.

By measuring the protein profiles both with SELDI-TOF MS and 2D-nanoLC-MS/MS we benefited of the advantages of two complementary methods. SELDI-TOF MS has the advantage to simultaneously measure parts of the serum proteome in a high-throughput fashion with relative simple sample preparation, high analytical sensitivity and high speed of data acquisition [34,35]. Although with 2D-nanoLC-MS/MS fewer samples can be measured simultaneously, this method has the advantage that it can identify the detected proteins immediately. Moreover, the protein detection by these two methods is complementary. With SELDI-TOF MS mainly measuring proteins in the 2 to 10 kDa mass range, many break-down products can be detected. Additionally, by measuring exact mass-to-charge ratios with SELDI-TOF MS, it is also possible to detect post-translational modified forms of proteins; for example proteins with additional amino acids or truncated forms. With 2D-nanoLC-MS/MS in combination with iTRAQ-labeling a higher selectivity is reached because of analysis of tryptic peptides with protein identification based on sequence information. This allows proteins with higher mass to be identified which cannot be detected with high sensitivity by SELDI-TOF MS.

## Conclusions

We detected several serum proteins that differed in concentration between women with asymptomatic breast cancer and matched healthy controls. For some of the proteins this may have been a chance finding, but C3a_desArg _and ITIH4 have previously also been found in relation with symptomatic breast cancer. Remarkably, high abundant, acute phase proteins, which we expected only to be detectable in symptomatic cancer cases, were also found to be significantly higher before diagnosis. Given that the currently identified proteins are high abundant, they are unlikely to be breast cancer specific, at least on their own. The fact however, that inflammatory processes are already present up to three years before diagnosis needs to be further investigated. For the search for specific tumor markers, we should take into account that these are low abundant, as it is typical for known circulating tumor markers to have low concentrations [36]. Using techniques that give insight into 'the deeper/low abundant proteome', e.g. by fractionation of the samples or depletion of a higher number of the most abundant proteins, which was already partially done in the 2Dnano-LC-MS/MS analysis, may help to find these low abundant and probably more specific tumor markers.

## Competing interests

The authors declare that they have no competing interests.

## Authors' contributions

AWJO, EJMK, CHvG and RCHV conducted the general design of the study. AWJO and MWG performed the protein profiling analysis. EJMK performed the 2D-nano-LC-MS/MS analysis with assistance of MHK, CHL and MCJ. AWJO, EJMK, CHvG and RCHV were involved in the data-analysis and drafted the manuscript. MHK, MWG, BAGJ, DEG, PHMP and JHB participated in editing and reviewing of the manuscript. All authors read and approved the final manuscript.

## Pre-publication history

The pre-publication history for this paper can be accessed here:

http://www.biomedcentral.com/1471-2407/11/381/prepub

## Supplementary Material

Additional file 1 **SELDI-TOF MS data collection**. Settings of the ProteinChip Reader, way of processing the spectra and settings for peak detection. Click here for file

Additional file 2 **2D-nanoLC-MS/MS analysis**. Details about the used columns and mobile phases, and about the acquisition and calibration. Click here for file

Additional file 3 **2D-nanoLC-MS/MS data analysis**. Details on search parameters for identification, and on data processing for quantification. Click here for file

Additional file 4 **Characteristics of the subset**. Characteristics of the subjects in the subset analyzed by 2D-nanoLC-MS/MS. Click here for file

Additional file 5 **Characteristics of the serum samples in the subset**. Characteristics of the serum samples in the subset analyzed by 2D-nanoLC-MS/MS. Click here for file
