# Supplementary material for: Searching for early breast cancer biomarkers by serum protein profiling of pre-diagnostic serum; a nested case-control study
Source: BMC Cancer. 2011 Aug 26;11:381. doi: 10.1186/1471-2407-11-381 (PMC3189190; doi:10.1186/1471-2407-11-381)
Supplement: Additional file 1 — SELDI-TOF MS data collection. Settings of the ProteinChip Reader, way of processing the spectra and settings for peak detection. [file 1471-2407-11-381-S1.PDF]

Additional file 1

SELDI-TOF MS data collection

With the PBS-IIC ProteinChip Reader (Bio-Rad Labs), 96 shots were fired on every spot, with a laser intensity of 153, and a detector sensitivity of 6. This was done after two warming shots per position, with a laser intensity of 155, which were not included in the final spectrum. Masses up to 160,000 Da were detected with an optimization range from 1,500 Da to 12,000 Da and with a focus at 8,000 Da. Mass to charge ratios (*m/z*s) were calibrated externally with the All-in-one standard peptide mixture (Bio-Rad Labs).

Spectra were first baseline subtracted, noise was estimated in the range from 2,000 to 160,000 Da, and spectra were normalized to the total ion current in the same range. Spectra with a normalization factor >2.00 or <0.50 were excluded from further analysis.

Peaks with a signal-to-noise ratio (S/N) greater than 5, and present in at least 20% of the spectra, were auto-detected in the first pass. In the second pass, peaks were detected with an S/N greater than 2, within a 0.3% mass window of the already detected peaks. Since not all peaks were auto-detected in each batch, all peaks auto-detected in at least one batch were subsequently manually detected in all batches. Thereafter, the BMW was applied on these user-detected peaks. Finally, the peaks with an S/N >2 in at least 50% of the spectra per batch were selected for further analysis.
