# Supplementary material for: Searching for early breast cancer biomarkers by serum protein profiling of pre-diagnostic serum; a nested case-control study
Source: BMC Cancer. 2011 Aug 26;11:381. doi: 10.1186/1471-2407-11-381 (PMC3189190; doi:10.1186/1471-2407-11-381)
Supplement: Additional file 2 — 2D-nanoLC-MS/MS analysis. Details about the used columns and mobile phases, and about the acquisition and calibration. [file 1471-2407-11-381-S2.PDF]

Additional file 2

2D-nanoLC-MS/MS analysis

In the first dimension peptides were separated on a strong cation-exchange (SCX) column (35 × 0.3 mm ZORBAX SCX Bioseries II, Agilent technologies) with mobile phase A (water with 3% acetonitrile and 0.1% formic acid) and B (0.5 M ammonium formate, 3% acetonitrile pH set to 3.5 using formic acid) using the capillary pump. The flow was set to 10 µl/min. The sample was eluted in 9 steps:;1% B, 1-6% B, 6-6.5% B, 6.5-10% B, 10-12.5% B, 12.5-15% B, 15-22% B, 22-30% B and 30-80% B (thus yielding a total of 9 fractions). The eluted peptides were trapped on a C_{18} trap column (5 µm 5×0.3 mm, ZORBAX; Agilent Technologies), the valve was then switched so that the nanoflow path was redirected through the trap-column and then onto a C_{18} analysis column (3.5 µm 150 mm × 100 µm, ZORBAX; Agilent Technologies). The mobile phases of the nanopump were A: water and B: acetonitrile both acidified with 0.1% formic acid. In each run a gradient from 5-40% B for 85 min, from 40-70% B in 5 min and then back to 5% B was applied in a flow rate of 400 nl/min.

Information dependent acquisitions (IDA) were performed with a total cycle time of 9 s including a TOF MS survey scan (1 s, *m/z* 300-1100), followed by acquiring of MS/MS spectra (2 s, *m/z* 100-2000) of the 4 most intense ions with charge state 2 to 5. Former target ions were excluded for 50 s and IDA were collected for 120 min for each fraction. The instrument was calibrated with CsI (*m/z* 132.9054) and the pentapeptide iPDI (*m/z* 829.5398).
