# Supplementary material for: Searching for early breast cancer biomarkers by serum protein profiling of pre-diagnostic serum; a nested case-control study
Source: BMC Cancer. 2011 Aug 26;11:381. doi: 10.1186/1471-2407-11-381 (PMC3189190; doi:10.1186/1471-2407-11-381)
Supplement: Additional file 3 — 2D-nanoLC-MS/MS data analysis. Details on search parameters for identification, and on data processing for quantification. [file 1471-2407-11-381-S3.PDF]

Additional file 3

2D-nanoLC-MS/MS data analysis

Search parameters were as follows: cystein modification, iodoacetamide; digestion, trypsin; search effort, thorough ID; instrument, QSTAR ESI; detected protein threshold, >1,3 (only proteins identified with at least 95% confidence). To compare expression levels in cases vs. controls tag114-labeled samples were normalized to the tag115-labeled samples and tag116 to tag117, respectively. Also, the ratios were corrected for unequal mixing of proteins when preparing the samples by dividing the ratios with the median average protein ratio calculated for each pair of an iTRAQ set (bias correction).
