# Supplementary material for: Searching for early breast cancer biomarkers by serum protein profiling of pre-diagnostic serum; a nested case-control study
Source: BMC Cancer. 2011 Aug 26;11:381. doi: 10.1186/1471-2407-11-381 (PMC3189190; doi:10.1186/1471-2407-11-381)
Supplement: Additional file 4 — Characteristics of the subset. Characteristics of the subjects in the subset analyzed by 2D-nanoLC-MS/MS. [file 1471-2407-11-381-S4.PDF]

Additional file 4

Characteristics of the subset

	**Cases** (*n*=20)	**Controls** (*n*=20)	**P-value**[††]
**Age at enrollment** (years)			
Mean (SD)	59.6 (6.0)	59.6 (6.1)	0.995
**BMI**			
Mean (SD)	26.8 (3.5)	26.5 (3.6)	0.764
Missing	1	-	
**Use of oral contraceptives,** *n* (%)			
No, but used to in the past	11 (55.0)	10 (50.0)	0.752
No, never	9 (45.0)	10 (50.0)	
**Duration of oral contraceptives use**[*] (years)			
Median (IQR)	10 (4-12)	4.5 (2.5-10.5)	0.272
**Use of HT,** *n* (%)			
No, but used to in the past	-	-	-
No, never	20 (100)	20 (100)	
**Ovariectomy,** *n* (%)			
Both ovaries removed	1 (5.0)	-	0.311
**Parity,** *n* (%)			
Nulliparous	2 (10.0)	-	0.147
**Number of children**[†]			
Median (IQR)	2.0 (2.0-3.0)	3.0 (2.0-3.8)	0.065
**Smoking,** *n* (%)			
No, but used to in the past	10 (50.0)	10 (50.0)	1.000
No, never	10 (50.0)	10 (50.0)	
**Pack-years smoking until stop date**[‡]			
Median (IQR)	3.1 (1.6-7.9)	7.2 (1.1-16.4)	0.536
Missing	1	1	
**Alcohol intake**[§] (g/day)			
Median (IQR)	5.1 (0.8-17.0)	3.4 (0.1-6.4)	0.234
**Use of medicines, minerals or vitamins**[#]**,** *n* (%)			
Yes	16 (80.0)	11 (55.0)	0.091
No	4 (20.0)	9 (45.0)	
**Minutes since last meal and/or drink**[**] (min)			
Median (IQR)	122 (97-160)	134 (87-145)	0.829

SD: Standard Deviation; BMI: Body Mass Index; IQR: Inter Quartile Range; HT: menopausal Hormone Therapy; [*] Among former oral contraceptives/HT users; [†] Among women with children; [‡] Among former smokers; [§] Energy-adjusted alcohol intake at enrollment; [#] in last week before blood collection; [**] at moment of blood collection; [††] Independent samples T test for age and BMI, Mann-Whitney U test for other continuous variables, and Pearson Chi-Square test for categorical variables.
