# Supplementary material for: Searching for early breast cancer biomarkers by serum protein profiling of pre-diagnostic serum; a nested case-control study
Source: BMC Cancer. 2011 Aug 26;11:381. doi: 10.1186/1471-2407-11-381 (PMC3189190; doi:10.1186/1471-2407-11-381)
Supplement: Additional file 5 — Characteristics of the serum samples in the subset. Characteristics of the serum samples in the subset analyzed by 2D-nanoLC-MS/MS. [file 1471-2407-11-381-S5.PDF]

Additional file 5

Characteristics of the serum samples in the subset

	Cases (*n*=20)	Controls (*n*=20)	P-value
**Serum sample storage duration*** (years)			
Mean (SD)	11.5 (1.1)	11.5 (1.1)	0.965§
**Hours in refrigerator**†			
Median (IQR)	21 (19-22)	22 (18-22)	0.978#
**Days at -86 °C**‡			
Median (IQR)	9 (5-21)	9 (5-21)	0.755#

SD: Standard Deviation; IQR: Inter Quartile Range; * Until date of experiment; † Between collection and centrifugation; ‡ Between centrifugation and storage at liquid nitrogen; § Independent samples T test; # Mann-Whitney U test.
